# Notch Sensitivity of Hydrogen-Charged 316L Stainless Steel: Experimental Insights into Mechanical Degradation and Fracture Mechanics

**DOI:** 10.3390/ma18061274

**Published:** 2025-03-13

**Authors:** Byeong-Kwan Hwang, Seung-Joo Cha, Hee-Tae Kim, Seung-Jun Lee, Jeong-Hyeon Kim, Jae-Myung Lee

**Affiliations:** 1 Dept. of Naval Architecture & Ocean Engineering, Pusan National Univ., Busan 46241, Republic of Korea; 2 Hydrogen Ship Technology Center, Pusan National Univ., Busan 46241, Republic of Korea

**Keywords:** hydrogen embrittlement, 316L stainless steel, hydrogen-assisted cracking, stress concentration, electrochemical hydrogen charging, mechanical degradation

## Abstract

Hydrogen is a promising eco-friendly energy source, but its embrittlement effect on structural materials remains a significant challenge. This study investigates the notch sensitivity of 316L stainless steel under in situ electrochemical hydrogen charging, with a focus on mechanical degradation and fracture behavior. By examining the influence of notch geometry and hydrogen exposure, this research highlights the role of stress concentration in hydrogen embrittlement. The findings contribute to understanding hydrogen-induced material failure, offering insights for both industry practitioners in the energy sector and academic researchers. This study also underscores the need for further research on hydrogen-resistant materials and structural safety considerations in hydrogen applications.

## 1. Introduction

Due to the increasing global demand for eco-friendly energy, hydrogen is being projected as an alternative energy source to traditional fossil fuels. Hydrogen can be produced from renewable energy sources such as wind and solar power. In addition, it is known as an environmentally friendly energy source with high energy density and almost no carbon emissions. Therefore, extensive research is being conducted on materials for storage and production technologies to utilize hydrogen [1]. Materials such as metals suffer from hydrogen embrittlement, which occurs when hydrogen diffuses into them, causing them to become brittle and fracture [2]. This not only significantly reduces the mechanical performance of the metal but also leads to unexpected breakage, posing a safety issue in hydrogen storage and transportation systems [3].

Among the materials generally used in hydrogen environments, austenite stainless steel has been applied due to its excellent corrosion resistance and mechanical properties [4]. 316L stainless steel has excellent weldability and grain boundary corrosion resistance and is mainly used due to its relatively high resistance to hydrogen embrittlement [5]. This is because austenite stainless steel has a face-centered cubic structure to prevent the diffusion of hydrogen atoms. However, despite these properties, 316L stainless steel suffers from hydrogen embrittlement problems [6]. Therefore, it is necessary to analyze the effect of hydrogen embrittlement on the 316L stainless steel to ensure the safe performance of the hydrogen system.

The susceptibility of the steel to hydrogen embrittlement is influenced by factors such as temperature [7], pressure [8], and geometric phenomena [9]. Accordingly, numerous researchers have investigated hydrogen embrittlement in 316L stainless steel through various experimental approaches, revealing the complex interactions of factors affecting mechanical properties.

Zhao et al. investigated the influence of grain refinement on hydrogen-induced delayed fracture in notched 316L stainless steel. Their findings demonstrated that finer grains enhance resistance to embrittlement by modifying hydrogen diffusion pathways and crack propagation behavior [10]. Song et al. examined the tensile properties of 316L stainless steel under high-pressure gaseous hydrogen. Their results indicated that hydrogen exposure significantly reduces fracture strength and alters fracture morphology, highlighting the detrimental effects of hydrogen on mechanical performance [11]. Beyond direct hydrogen exposure, manufacturing methods and material processing also play a crucial role in embrittlement susceptibility. Nietzke et al. studied the embrittlement behavior of additively manufactured 316L stainless steel, providing evidence that different production techniques influence hydrogen diffusion characteristics and overall mechanical integrity [12]. Similarly, Álvarez et al. explored the effects of post-processing and temperature, revealing that embrittlement becomes more pronounced at lower temperatures, emphasizing the necessity of optimized thermal treatment [13]. Environmental conditions further contribute to hydrogen embrittlement susceptibility. Komatsu et al. analyzed the formation of hydrogen-induced vacancies in 316L stainless steel under varying straining temperatures. Their study confirmed that localized strain regions act as critical initiation sites for embrittlement, leading to structural degradation [14]. Meanwhile, Huang et al. examined the effects of laser peening on the tensile properties of 316L stainless steel in a hydrogen-rich environment. Their research demonstrated that laser peening enhances resistance to embrittlement by inducing compressive residual stress and refining the grain structure [15]. Additionally, Nguyen et al. conducted experimental studies on the hydrogen compatibility of 316L stainless steel at cryogenic temperatures. Their findings highlighted that low temperatures significantly exacerbate embrittlement susceptibility, reinforcing the crucial role of temperature in hydrogen-induced material degradation [16].

Previous studies have extensively analyzed the effects of external factors such as hydrogen charging conditions, stress levels, and environmental influences on the hydrogen embrittlement of 316L stainless steel. However, the influence of geometric features, particularly notches, on hydrogen embrittlement susceptibility has not been systematically evaluated. Notch geometry plays a critical role, as it induces stress concentration, accelerating hydrogen diffusion and trapping, which in turn leads to material degradation [17]. As notch severity increases, the local hydrogen fugacity near the notch also rises, further reducing notch strength [18]. Therefore, understanding the hydrogen embrittlement behavior of notched 316L stainless steel is essential for assessing material failure risks.

To address this, this study investigates the mechanical properties of 316L stainless steel with various notch geometries under in situ electrochemical hydrogen charging. The specimens feature notch root radii of 0.1, 1, 3, and 10 mm and are exposed to current densities ranging from 1 to 50 mA/cm^2^. In situ electrochemical hydrogen charging ensures a uniform distribution of hydrogen within the specimens [19]. By examining the interplay between notch geometry, hydrogen concentration, and mechanical degradation, this study aims to shed light on the mechanisms of hydrogen-assisted cracking in notched components. The findings are expected to provide critical guidelines for designing hydrogen-resistant materials and structures.

The structure of this paper is as follows. Section 2 describes the experimental setup, including specimen preparation, electrochemical hydrogen charging, and mechanical testing procedures. Section 3 presents and discusses experimental results, focusing on the effects of notch geometry and hydrogen charging conditions on mechanical degradation and embrittlement susceptibility. This section also includes stress–strain analysis, hydrogen concentration measurements, and fractographic observations to analyze the mechanisms of hydrogen-assisted cracking. Finally, Section 4 summarizes the key findings, discusses their implications for the design of hydrogen-resistant materials and structural safety, and proposes future research directions for further analysis of hydrogen embrittlement in notched components.

## 2. Experimental Preparations

### 2.1. Experimental Setup and Specimen Preparation

**Figure 1:**
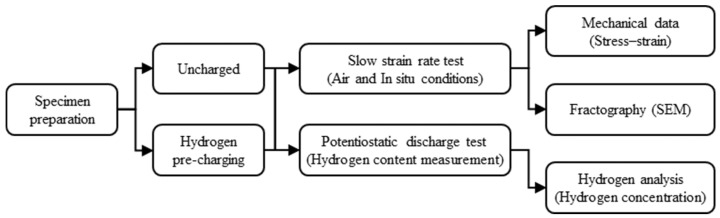
Flowchart of notch sensitivity testing in hydrogen-charged 316L stainless steel.

This study investigates the notch sensitivity of hydrogen-charged 316L stainless steel through a series of experimental procedures. Figure 1 presents a flowchart outlining the experimental workflow, which includes specimen preparation, hydrogen charging, mechanical testing, and subsequent analyses.

**Figure 2:**
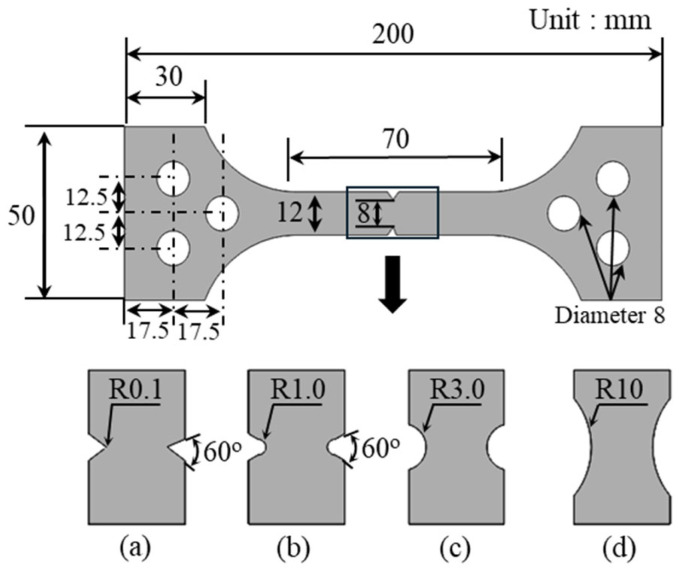
Tensile specimens: notch radius (**a**) 0.1 mm, (**b**) 1.0 mm, (**c**) 3.0 mm, and (**d**) 10 mm.

The first step in this process is specimen preparation. The chemical composition of the 316L stainless steel used in this study was analyzed using a spark emission spectrometer, which provides precise elemental analysis of metals. The analysis included key alloying elements such as C, Si, Mn, P, S, Ni, Cr, Mo, Cu, V, and W, ensuring accurate performance evaluation. The detailed chemical composition is presented in Table 1. To ensure uniform microstructure and mechanical properties, the material underwent a series of cold rolling and annealing processes. After these treatments, the material was machined into specimens with specific notch root radius of 0.1, 1, 3, and 10 mm. Each specimen was prepared in a plate form with a final thickness of 2 mm. All test specimens were fabricated with a notch width of 8 mm and a gauge length of 50 mm. Figure 2 illustrates the dimensions of the specimens.

### 2.2. SSRT Under Cathodic Charging

**Figure 3:**
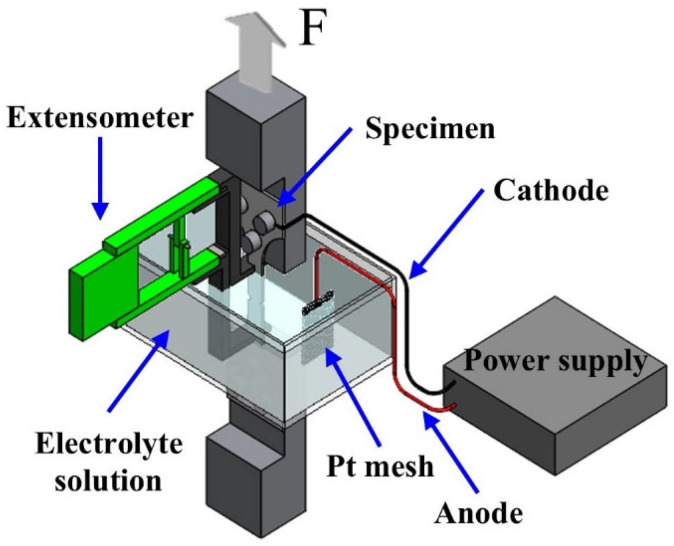
Schematic diagram of the in situ electrochemical SSRT test setup.

Figure 3 shows a schematic diagram of the in situ electrochemical SSRT test conducted in this study. To evaluate the susceptibility to hydrogen embrittlement, hydrogen was electrochemically charged into the specimens at room temperature using current densities of 1, 2, 5, 10, 30, and 50 mA/cm^2^. The electrolyte solution used for the electrochemical charging process was composed of 3% NaCl and 0.3% NH_4_SCN. In this setup, the specimens were used as the working electrodes, while platinum served as the counter electrode.

Tensile tests were performed on specimens without hydrogen charging and on specimens charged with hydrogen via in situ electrochemical methods. The strain rate for all specimens was 3 × 10^−5^/s. For the in situ tests, the tensile tests were conducted after approximately 72 h of hydrogen charging to ensure that the hydrogen was sufficiently charged and diffused into the specimens. To maintain a continuous hydrogen exposure environment during the tensile tests, hydrogen charging was continued. Since it was not possible to directly connect an extensometer in the in situ tests, a specially designed drop-down extensometer, as referenced from the literature, was applied [20]. To ensure the repeatability and reliability of the experimental results, each test condition was repeated three times for all scenarios, and the average values were calculated. The study was conducted with current densities of 1, 2, 5, 10, 30, and 50 mA/cm^2^ and notch root radii of 0.1, 1, 3, and 10 mm. The hydrogen-charged specimens experience significant loss of ductility and a decrease in notch strength [21]. Therefore, for each notch test, the hydrogen embrittlement susceptibility index was calculated using the test-derived average values as Relative Notch Tensile Strength (*RNTS*), as shown in Equation (1), and Relative Elongation (*REL*), as shown in Equation (2).

(1) RNTS=NTS in Hydrogen/NTS in Air

(2) REL=Elongation in Hydrogen/Elongation in Air

### 2.3. Measurement of Hydrogen Concentration

To measure hydrogen concentration, the current generated by the electrochemical oxidation method is analyzed. The specimens used in this analysis are 5 mm × 5 mm × 2 mm in size and were pre-charged with the same current densities (1, 2, 5, 10, 30, and 50 mA/cm^2^) as the tensile test specimens. After completing the hydrogen charging, the specimens were rinsed with 0.1 mol/L NaOH and then immersed in a deoxygenated NaOH solution of the same concentration. Oxygen removal from the solution was performed by purging with nitrogen gas for 1 h. The specimen was used as the working electrode, a platinum sheet as the counter electrode, and a mercury/mercury oxide (Hg/HgO) electrode as the reference electrode. The experiment was conducted at a constant potential of +300 mV (vs. Hg/HgO) for 1 h [22]. Current changes were recorded to produce a current-time curve, which was then integrated to calculate the hydrogen concentration. The hydrogen concentration at each current density was determined by subtracting the background current from the current observed in the hydrogen-charged specimens [23]. To ensure measurement reliability and reproducibility, each measurement was repeated three times, and the average values along with the standard deviation were calculated. The study was conducted with current densities of 1, 2, 5, 10, 30, and 50 mA/cm^2^.

### 2.4. Fractographic Feature Observations

After the completion of the SSRT, an analysis of the fracture surfaces was conducted to evaluate the effects of hydrogen charging. This analysis involved a comparison between specimens that had not undergone hydrogen charging and those that had. Scanning Electron Microscopy (SEM) was utilized for this analysis, specifically using the SUPRA25 model equipped with a high-resolution, wide-field electron optics system.

## 3. Result and Discussion

### 3.1. SSRT Results

**Figure 4:**
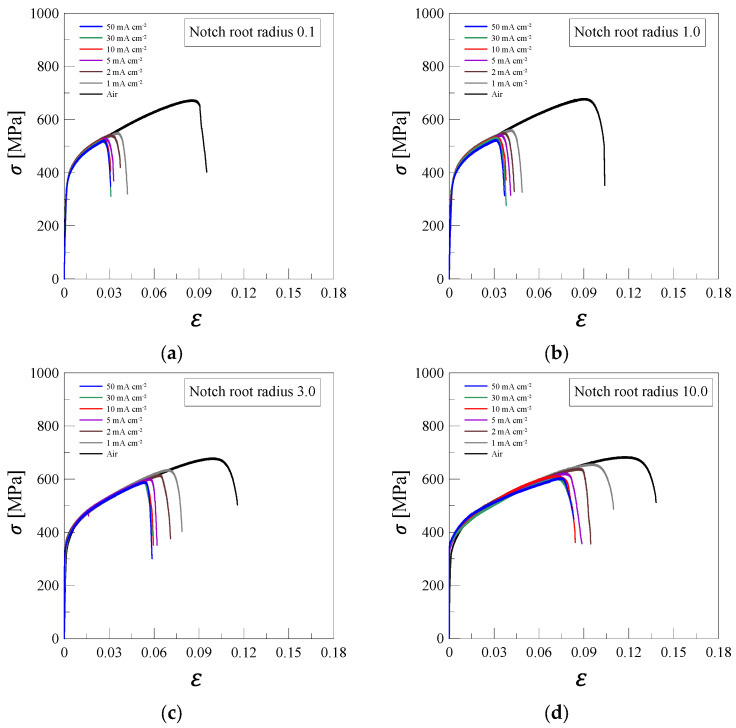
Stress–strain curves of 316L stainless steel under various notch root radius and hydrogen charging conditions: (**a**) 0.1, (**b**) 1.0, (**c**) 3.0, and (**d**) 10.0.

The stress–strain curves of 316L stainless steel, subjected to SSRT tests under various hydrogen charging conditions, are shown in Figure 4. This figure illustrates the impact of different notch root radii (0.1, 1.0, 3.0, 10.0) and current densities (1 to 50 mA/cm^2^) on the mechanical performance of the material. Stress and strain are calculated using the measured force, as shown in Equation (3), and the measured displacement, as shown in Equation (4):

(3) σ=F/A

(4) $$\varepsilon =∆L/L_{0}$$

where F is the applied force, A is the initial cross-sectional area of the specimen, ∆L is the measured displacement, and $L_{0}$ is the initial gauge length of the specimen. The critical force ($F_{C}$), notch strength ($\sigma_{N}$), critical displacement (${∆L}_{C}$), and elongation ($\varepsilon_{f}$) calculated through SSRT are presented in Table 2. The critical force refers to the maximum applied force before failure or significant plastic deformation occurs in the notched specimen. The notch strength represents the ultimate tensile strength considering the notch effect and is determined by dividing the critical force by the initial cross-sectional area at the notch. The critical displacement corresponds to the displacement at which the specimen reaches its critical force before failure. Lastly, the elongation indicates the total strain at failure, reflecting the overall elongation of the specimen before complete fracture.

The experimental results showed that hydrogen embrittlement occurred in the plastic region rather than in the elastic region in all specimens. This is likely due to hydrogen-enhanced dislocation movement and microstructural changes that take place during plastic deformation [24]. As the hydrogen charging current density increases, both notch strength and strain of failure decrease. This occurs because a higher current density results in more hydrogen atoms being introduced into the material, increasing the likelihood of hydrogen interacting with internal defects such as dislocations, grain boundaries, and voids. These interactions facilitate crack initiation and propagation, weakening the material and leading to premature failure [25]. However, the stress–strain curves of hydrogen-charged specimens show similar trends at current densities exceeding 10 mA/cm^2^. This suggests that a critical threshold for hydrogen embrittlement has been reached. Beyond this threshold, further increases in current density do not significantly alter the embrittlement effect, indicating that hydrogen saturation has occurred at grain boundaries and dislocations [26].

A quantitative analysis of Table 2 supports these observations. At a notch root radius of 0.1 mm, the notch strength decreased from 675 MPa at 0 mA/cm^2^ to 525 MPa at 10 mA/cm^2^, while the elongation dropped from 0.095 to 0.031. Similar trends were observed for other notch radii, confirming that an increase in hydrogen charging current density leads to a reduction in mechanical properties. However, at current densities above 10 mA/cm^2^, the notch strength values stabilize, suggesting that hydrogen embrittlement effects reach a saturation point.

Another key factor influencing susceptibility to hydrogen embrittlement is the notch root radius [27]. A smaller notch root radius increases local stress concentration, which enhances hydrogen diffusion and trapping [28]. As seen in Table 2, specimens with a notch root radius of 0.1 mm exhibited a notch strength of only 522 MPa at 50 mA/cm^2^, whereas those with a 10.0 mm notch root radius maintained a significantly higher strength of 612 MPa. Similarly, the elongation was only 0.031 for the 0.1 mm notch at 50 mA/cm^2^, while the 10.0 mm notch remained 0.083 under the same conditions. This confirms that larger notch root radii result in a more even stress distribution, reducing localized hydrogen embrittlement effects and improving overall mechanical stability [29].

In conclusion, this experiment confirms that the degradation of mechanical performance caused by hydrogen charging is significantly influenced by both the notch root radius and charging current density. Hydrogen embrittlement is more severe at smaller notch radii and higher current densities. However, beyond a critical current density of 10 mA/cm^2^, the embrittlement effect appears to saturate, indicating that hydrogen trapping and saturation at grain boundaries and dislocations play a dominant role in determining the material’s mechanical properties.

### 3.2. Hydrogen Embrittlement Susceptibility Index

**Figure 5:**
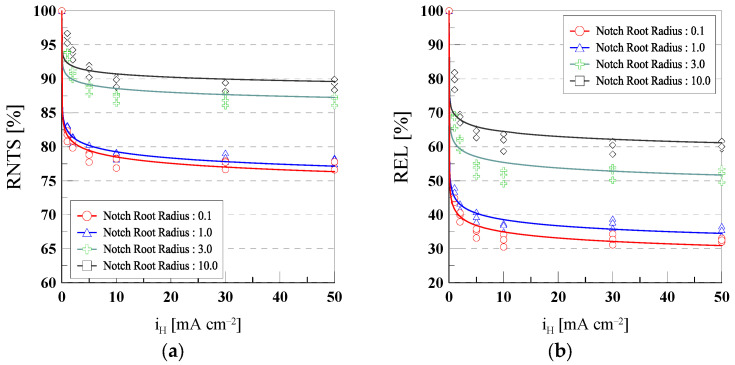
(**a**) RNTS and (**b**) REL as a function of current density for different notch root radius specimens.

Figure 5 shows the hydrogen embrittlement sensitivity of 316L stainless steel as a function of current density, presented in terms of RNTS and REL relative to the notch root radius. The experimental results were fitted using Equation (5):

(5) $$HE\;index=m\;\times \;i_{H}^{n}$$

where HE index is the Hydrogen Embrittlement index, m is a factor, $i_{H}$ is the current density, and n is the exponent. Table 3 presents the parameter values for *m* and n for RNTS and REL.

Analysis of the variations in RNTS and REL with respect to current density revealed a sensitivity difference based on notch root radius. Specimens with smaller notch root radii (0.1 and 1.0) exhibited a pronounced decrease in RNTS and REL under low current density conditions (1 mA/cm^2^), significantly influenced by electrochemical hydrogen embrittlement.

Specifically, for a radius of 0.1, RNTS dropped to approximately 81.7% at 1 mA/cm^2^ and stabilized at around 77.4% as the current density increased to the 10–50 mA/cm^2^ range. REL also showed a sharp decrease from 44.4% to 32.5% within the same current density range. Similarly, for a radius of 1.0, RNTS decreased from 82.7% at 1 mA/cm^2^ to 77.2%, while REL dropped from 46.9% to 35.4%. This indicates that smaller radii experience greater localized stress concentration, making them more susceptible to the effects of hydrogen.

In contrast, specimens with larger notch root radii (3.0 and 10.0) showed relatively gradual decreases in RNTS and REL with increasing current density. For a radius of 3.0, RNTS decreased from approximately 93.5% at 1 mA/cm^2^ to 86.9% at 50 mA/cm^2^, while REL declined from 68.0% to 51.8%. For a radius of 10.0, RNTS decreased from about 96.0% at 1 mA/cm^2^ to 88.4%, and REL dropped from 79.4% to 60.3%.

This more gradual reduction can be attributed to the relatively uniform stress distribution in larger radii, which provides structural stability and reduces susceptibility to hydrogen.

In conclusion, smaller notch root radii exhibit higher sensitivity to changes in RNTS and REL with increasing current density due to localized stress concentration, with stabilization occurring in the 10–50 mA/cm^2^ range. In contrast, larger notch root radius exhibits lower sensitivity, showing a more gradual reduction due to their structural stability.

### 3.3. Hydrogen Concentration

**Figure 6:**
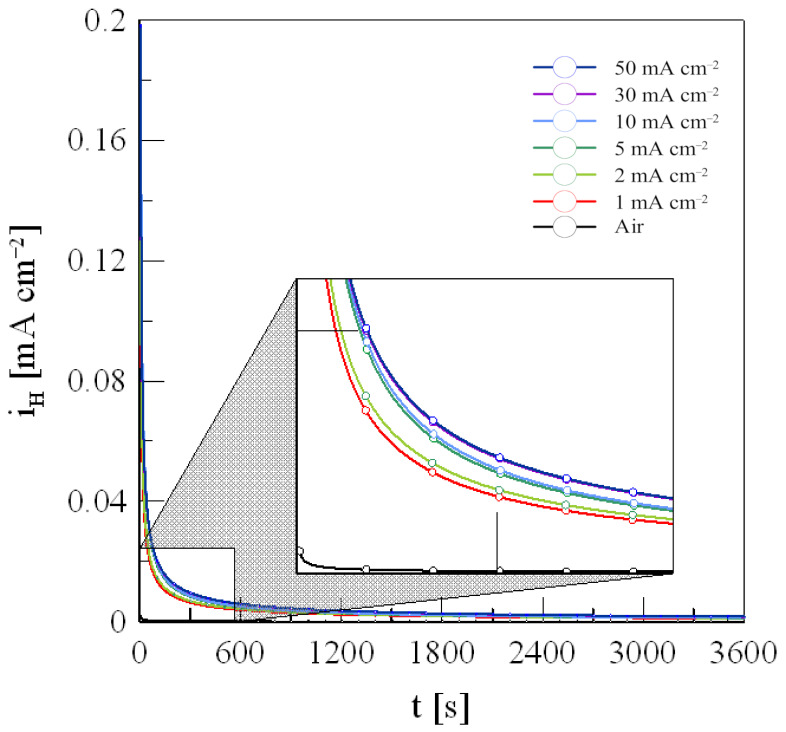
Discharge current density of 316L stainless steel charged with hydrogen at various current densities.

Figure 6 shows the current-time curves for different charging current densities in response to variations in current density during anodization. The experimental results show a rapid decrease in current density up to 300 s for all current densities, followed by a slower decline. This behavior is due to the absorption of most hydrogen atoms near the metal surface during electrochemical hydrogen charging. Consequently, electrochemical hydrogen charging has a significant impact on the metal surface, as noted by previous studies [30].

The amount of hydrogen released from the metal is calculated by integrating the anodic oxidation current density over time, as shown in Equation (6). Based on this, the diffusible hydrogen concentration (*C_H_*) in steel can be calculated using Equation (7) [31].

(6) $$Q_{H}=\int_{0}^{\tau_{\mathrm{dis}}}(i_{H}-i_{Air})d\tau$$

(7) $$C_{H}=Q_{H}/zF_{A}V$$

where $Q_{H}$ is the charge of diffusible hydrogen, z is the valence number for hydrogen, which is 1, $F_{A}$ is the faraday constant (96,487 C/mol), and V is the effective volume.

**Figure 7:**
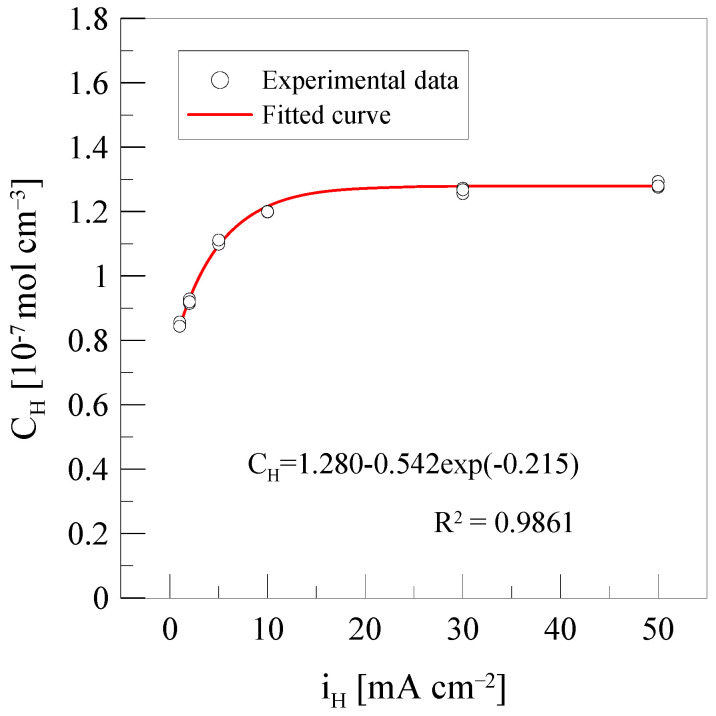
Total hydrogen concentration after charging at various current densities.

Figure 7 shows the hydrogen concentration over a current density range of 1 to 50 mA/cm^2^. The relationship between hydrogen concentration and current density can be expressed by the following Equation (8) [31].

(8) $$C_{H}=C_{0}-A_{0}exp(R_{0}i_{H})$$

where $C_{0}$ represents the saturation level of hydrogen concentration, $A_{0}$ and $R_{0}$ are the sensitivity coefficient and exponent for hydrogen traps, respectively. $C_{0}$, $A_{0}$, and $R_{0}$ are each related to the number of hydrogen traps and the ability of these traps to capture hydrogen. The experimental results indicate that as the current density increases, the hydrogen concentration also increases. This is because a higher current density leads to more hydrogen accumulating on the metal surface, resulting in an increased amount of hydrogen charging [32]. Therefore, at lower current densities, fewer hydrogen atoms are absorbed, limiting the interaction with hydrogen traps. However, beyond 30 mA/cm^2^, the increase in hydrogen concentration becomes nonlinear, indicating that the hydrogen traps have reached their saturation point. This trend is observed in Table 4, where the increase in total hydrogen concentration slows down at higher current densities. This phenomenon occurs because the number of available hydrogen traps in the material is limited. Once all traps are occupied, additional hydrogen atoms can no longer be effectively stored. When hydrogen absorption reaches saturation, further uptake ceases, and hydrogen permeation and diffusion become restricted [33]. Consequently, at higher current densities, the material reaches saturation more quickly, leading to a decrease in hydrogen absorption efficiency.

**Figure 8:**
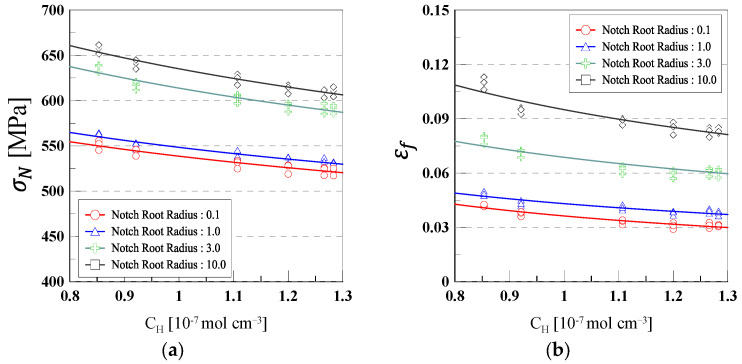
Variations in notch strength and elongation as a function of total hydrogen concentration for different notch root radii: (**a**) notch strength, (**b**) elongation.

Finally, using Equations (9) and (10), the relationships between total hydrogen concentration and notch strength as well as elongation are presented in Figure 8. These equations describe the influence of hydrogen concentration on the mechanical properties of 316L stainless steel, showing a quantitative correlation between hydrogen uptake and degradation in mechanical performance.

The corresponding regression parameters, including the coefficient values (m, n) and coefficient of determination (R^2^) for notch strength and elongation, are summarized in Table 5. These data provide an assessment of the impact of hydrogen concentration at different notch root radii (0.1, 1.0, 3.0, and 10.0 mm), indicating how localized stress concentration influences material degradation under hydrogen exposure.

The regression analysis demonstrates that as the hydrogen concentration increases, both notch strength and elongation exhibit a decreasing trend, with a more pronounced effect at smaller notch root radius due to higher localized stress. This confirms that the extent of hydrogen embrittlement is significantly affected by the notch geometry and associated stress distribution.

(9) $$\sigma_{N}=\;m\;\times \;C_{H}^{n}$$

(10) $$\varepsilon_{f}=\;m\;\times \;C_{H}^{n}$$

### 3.4. Fractographic Analysis of the SSRT Specimens

**Figure 9:**
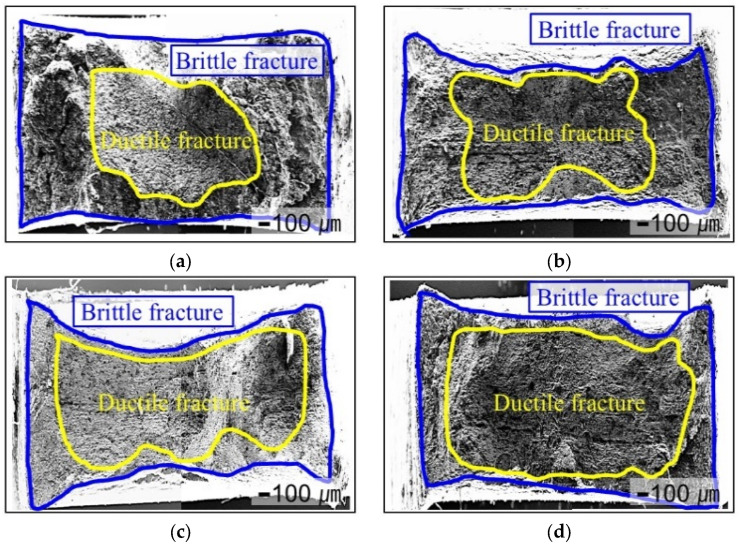
Representative fractured surfaces of various notch radii under a hydrogen environment: (**a**) 0.1, (**b**) 1.0, (**c**) 3.0, and (**d**) 10.0.

Figure 9 shows the fracture surfaces of specimens electrochemically charged with hydrogen at 10 mA/cm^2^ after the SSRT test, depending on the notch radius. In the hydrogen-charged specimens, ductile fracture was observed in the central region, whereas brittle fracture was predominantly concentrated at the upper and lower sections as well as the left and right edges of the specimen. This phenomenon is believed to be closely related to the tendency of hydrogen to accumulate at specific locations [34].

Furthermore, as the notch radius decreased, the tendency for brittle fracture increased. This can be attributed to the rise in stress concentration as the notch radius decreases, which leads to intensified local hydrogen accumulation and consequently promotes a brittle fracture.

**Figure 10:**
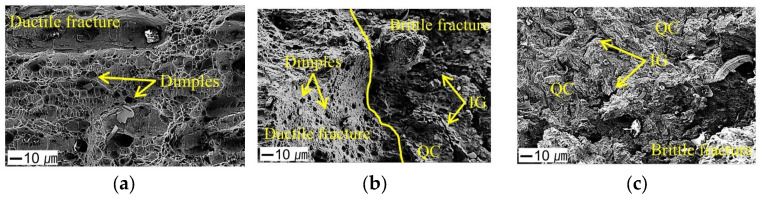
Representative fracture characteristics in different regions: (**a**) ductile fracture, (**b**) transition fracture, and (**c**) brittle fracture.

Figure 10 presents magnified images of microstructural features observed in each region to further clarify this fracture behavior. In the central region, where ductile fracture occurred (Figure 10a), dimples formed by the coalescence of microvoids were widely distributed. However, in the region where a brittle fracture was observed (Figure 10c), both intergranular (IG) and quasi-cleavage (QC) fracture features were present. This suggests that hydrogen increases microstructural susceptibility to embrittlement and promotes localized crack growth during deformation [35]. In the edge region where hydrogen accumulated intensively, cracks propagated in a brittle manner, which is considered a result of increased hydrogen-induced embrittlement and mechanical stress concentration [36].

Additionally, as seen in Figure 10b, in the mixed fracture region, both dimple structures and brittle fracture surfaces coexisted. This indicates that fracture modes can change depending on the local hydrogen concentration and stress distribution.

Ultimately, hydrogen charging accelerates the formation and growth of microcracks, altering the fracture behavior of the specimens. In hydrogen-affected specimens, the number of cracks increased, and ductile deformation on the fracture surface was reduced. This is likely due to hydrogen hindering dislocation movement and inducing localized brittle cracking, thereby increasing the contribution of brittle fracture mechanisms over conventional ductile fracture mechanisms [37].

## 4. Conclusions

In this study, the hydrogen embrittlement behavior of 316L stainless steel with various notch geometries was investigated under in situ electrochemical hydrogen charging conditions at different current densities ranging from 1 to 50 mA/cm^2^. The findings are summarized as follows:

1. The hydrogen embrittlement of 316L stainless steel was mainly influenced by the notch radius and current density. As the current density increased, both tensile strength and elongation tended to decrease. This was attributed to the increased diffusion of hydrogen into the material at higher current densities, which promoted crack initiation and propagation through interactions with dislocations, grain boundaries, and microvoids.
2. Experimental results indicated that the hydrogen embrittlement effect tended to saturate at current densities of 10 mA/cm^2^ or higher. This suggests that hydrogen saturation within grain boundaries and dislocation structures limits the additional impact of hydrogen charging on the mechanical properties.
3. As the notch radius decreased (0.1 mm and 1.0 mm), local stress concentration increased, promoting hydrogen diffusion and trapping, which led to a significant reduction in RNTS and REL. In contrast, specimens with larger notch radii (3.0 mm and 10.0 mm) exhibited a more uniform stress distribution, resulting in relatively lower susceptibility to hydrogen embrittlement.
4. As the current density increased, the hydrogen concentration inside the specimen also increased, showing a nonlinear increase at current densities above 30 mA/cm^2^. This indicates that additional hydrogen absorption is limited due to the saturation of hydrogen trapping sites, leading to a slowdown in changes in mechanical properties beyond a certain current density.
5. In the hydrogen-charged specimens, a mixed fracture mode of ductile fracture (dimples) and brittle fracture (quasi-cleavage and intergranular fracture) was observed. Notably, specimens with smaller notches exhibited a higher tendency for brittle fractures. This suggests that local hydrogen accumulation and stress concentration are key factors in promoting brittle cracking.

The findings of this study have significant implications for industries reliant on hydrogen-compatible materials, particularly in energy, hydrogen storage, fuel cells, and offshore/marine applications. The results can guide the design of high-pressure hydrogen storage tanks, fuel cell components, and offshore pipelines by providing insights into the effects of notch geometry and hydrogen concentration on mechanical properties. This knowledge is crucial for improving the safety and durability of hydrogen-based infrastructures, such as pressure vessels and aerospace components. The observed saturation effect at high current densities highlights design limitations for hydrogen-resistant stainless steels, informing future material development and safety protocols.

However, further research is needed to assess the long-term effects of hydrogen exposure, including fatigue and creep behavior, and to evaluate the impact of environmental factors like marine and cryogenic conditions on embrittlement susceptibility. Future studies should focus on prolonged hydrogen exposure and its effects on fatigue life and creep resistance, as well as the susceptibility of 316L stainless steel to embrittlement in harsh environments. These insights will be critical for advancing the safety and reliability of hydrogen storage and transportation systems in challenging conditions.

## Figures and Tables

**Table 1:** The chemical composition [wt %] of test specimen.

Alloys	C	Si	Mn	P	S	Ni	Cr	Mo
Type 316L	0.023	0.51	0.93	0.041	0.003	10.09	16.94	2.02

**Table 2:** Critical force, notch strength, critical displacement, and elongation of 316L stainless steel under various notch root radius and hydrogen charging conditions.

Notch Root Radius	Current Density [ma cm^−2^]	$F_{C}$ [N]	$\sigma_{N}$ [MPa]	${∆L}_{C}$ [mm]	$\varepsilon_{f}$
0.1	0	10,794 ± 55	675 ± 3	4.26 ± 0.11	0.095 ± 0.0030
	1	8818 ± 83	551 ± 5	1.78 ± 0.08	0.042 ± 0.0006
	2	8699 ± 65	544 ± 4	1.52 ± 0.02	0.037 ± 0.0013
	5	8481 ± 74	530 ± 4	1.34 ± 0.03	0.033 ± 0.0014
	10	8403 ± 84	525 ± 5	1.25 ± 0.08	0.031 ± 0.0018
	30	8363 ± 70	523 ± 4	1.25 ± 0.06	0.031 ± 0.0015
	50	8358 ± 68	522 ± 4	1.25 ± 0.05	0.031 ± 0.0005
1.0	0	10,870 ± 49	679 ± 3	4.50 ± 0.11	0.104 ± 0.0018
	1	8992 ± 63	562 ± 4	2.06 ± 0.08	0.049 ± 0.0012
	2	8816 ± 65	551 ± 4	1.84 ± 0.06	0.043 ± 0.0015
	5	8688 ± 62	543 ± 4	1.73 ± 0.07	0.041 ± 0.0015
	10	8566 ± 63	535 ± 4	1.55 ± 0.08	0.038 ± 0.0013
	30	8527 ± 70	533 ± 4	1.55 ± 0.07	0.039 ± 0.0014
	50	8396 ± 72	530 ± 5	1.56 ± 0.06	0.038 ± 0.0014
3.0	0	10,880 ± 63	680 ± 4	4.99 ± 0.14	0.116 ± 0.0030
	1	10,178 ± 68	636 ± 4	3.47 ± 0.09	0.079 ± 0.0024
	2	9865 ± 74	617 ± 5	3.16 ± 0.10	0.071 ± 0.0021
	5	9634 ± 77	602 ± 5	2.85 ± 0.09	0.062 ± 0.0023
	10	9487 ± 78	593 ± 5	2.69 ± 0.10	0.059 ± 0.0023
	30	9451 ± 91	591 ± 6	2.68 ± 0.09	0.061 ± 0.0022
	50	9452 ± 77	591 ± 5	2.70 ± 0.08	0.060 ± 0.0023
10.0	0	10,952 ± 70	684 ± 4	5.82 ± 0.12	0.138 ± 0.0035
	1	10,510 ± 80	657 ± 5	4.78 ± 0.15	0.110 ± 0.0033
	2	10,246 ± 82	640 ± 5	4.26 ± 0.11	0.095 ± 0.0018
	5	9965 ± 92	623 ± 6	3.86 ± 0.12	0.089 ± 0.0019
	10	9810 ± 81	613 ± 5	3.55 ± 0.09	0.084 ± 0.0035
	30	9740 ± 80	609 ± 5	3.54 ± 0.08	0.082 ± 0.0027
	50	9762 ± 87	612 ± 5	3.55 ± 0.08	0.083 ± 0.0015

**Table 3:** Changes in (a) RNTS and (b) REL as a function of current density.

Notch Root Radius	RNTS			REL		
	m	n	R2	m	n	R2
0.1	78.06	−0.021	0.93	49.16	−0.143	0.80
1.0	84.60	−0.035	0.85	64.49	−0.209	0.77
3.0	94.53	−0.033	0.96	79.03	−0.182	0.85
10.0	95.60	−0.034	0.88	82.99	−0.143	0.83

**Table 4:** Total hydrogen concentration at different charging current densities.

Sample	Total Hydrogen Concentration [10^−7^ mol cm^−3^]					
	1 mA cm^−2^	2 mA cm^−2^	5 mA cm^−2^	10 mA cm^−2^	30 mA cm^−2^	50 mA cm^−2^
1	0.844	0.920	1.112	1.200	1.268	1.280
2	0.856	0.929	1.110	1.201	1.256	1.276
3	0.857	0.915	1.099	1.199	1.273	1.294
Average	0.853	0.921	1.107	1.200	1.266	1.283
Standard deviation	0.006	0.006	0.006	0.001	0.007	0.008

**Table 5:** Regression coefficients for notch strength and elongation as a function of total hydrogen concentration at different notch root radii.

Notch Root Radius	$\sigma_{N}$			$\varepsilon_{f}$		
	*m*	n	R^2^	*m*	n	R^2^
0.1	538.58	−0.131	0.99	0.0363	−0.737	0.95
1.0	548.39	−0.132	0.97	0.0431	−0.570	0.91
3.0	613.77	−0.17	0.95	0.0687	−0.540	0.93
10.0	635.11	−0.177	0.98	0.095	−0.598	0.89

## Data Availability

The original contributions presented in this study are included in the article, further inquiries can be directed to the corresponding author.

## Abbreviations/Nomenclature

The following abbreviations are used in this manuscript:

RNTS	Relative Notch Tensile Strength
REL	Relative Elongation
SEM	Scanning Electron Microscopy
HE index	Hydrogen Embrittlement index
IG	Intergranular
QC	Quasi-Cleavage
σ	Stress
ε	Strain
F	Applied force
A	Initial cross-sectional area of the specimen
∆L	Measured displacement
$L_{0}$	Initial gauge length of the specimen
$F_{C}$	Critical force
$\sigma_{N}$	Notch strength
${∆L}_{C}$	Critical displacement
$\varepsilon_{f}$	Elongation
m, n	Fitted parameters
$i_{H}$	Current density
$Q_{H}$	Charge of diffusible hydrogen
z	Valence number for hydrogen
$F_{A}$	Faraday constant
V	Effective volume
$C_{H}$	Total hydrogen concentration
$C_{0}$	Saturation level of hydrogen concentration
$A_{0}$ $,\;R_{0}$	Sensitivity coefficient and exponent for hydrogen traps
